# Co-occurrence of planktonic bacteria and archaea affects their biogeographic patterns in China’s coastal wetlands

**DOI:** 10.1186/s40793-021-00388-9

**Published:** 2021-10-19

**Authors:** Baoli Wang, Na Liu, Meiling Yang, Lijia Wang, Xia Liang, Cong-Qiang Liu

**Affiliations:** 1 Institute of Surface-Earth System Science, School of Earth System Science, Tianjin University, Tianjin, 300072 China; 2 Critical Zone Observatory of Bohai Coastal Region, Tianjin University, Tianjin, 300072 China; 3 State Key Laboratory of Estuarine and Coastal Research, East China Normal University, Shanghai, 200244 China

**Keywords:** Biogeography, Planktonic bacteria and archaea, Co-occurrence, Coastal wetlands

## Abstract

Planktonic bacteria and archaea play a key role in maintaining ecological functions in aquatic ecosystems; however, their biogeographic patterns and underlying mechanisms have not been well known in coastal wetlands including multiple types and at a large space scale. Therefore, planktonic bacteria and archaea and related environmental factors were investigated in twenty-one wetlands along China’s coast to understand the above concerns. The results indicated that planktonic bacteria had different biogeographic pattern from planktonic archaea, and both patterns were not dependent on the wetland's types. Deterministic selection shapes the former’s community structure, whereas stochastic processes regulate the latter’s, being consistent with the fact that planktonic archaea have a larger niche breadth than planktonic bacteria. Planktonic bacteria and archaea co-occur, and their co-occurrence rather than salinity is more important in shaping their community structure although salinity is found to be a main environmental deterministic factor in the coastal wetland waters. This study highlights the role of planktonic bacteria-archaea co-occurrence on their biogeographic patterns, and thus provides a new insight into studying underlying mechanisms of microbial biogeography in coastal wetlands.

**Supplementary Information:**

The online version contains supplementary material available at 10.1186/s40793-021-00388-9.

## Background

Understanding microbial biogeographic patterns is a fundamental task of aquatic microbial ecology [1–6]. The biogeographic studies on aquatic microorganisms have experienced from arguing whether there are biogeographic patterns to concerning community assembly mechanisms [7–9]. Synchronously, the highlight is from a distance-decay relationship to a relative influence of deterministic and stochastic processes on present-day distribution patterns [10–12]. Deterministic processes are based on traditional niche theory including biotic interactions and environmental filtering, while stochastic processes are based on neutral theory including drift and extinction [13, 14]. Although four basic conceptual processes (i.e., selection, drift, dispersal, and mutation) are well known to create and maintain microbial biogeographic patterns [15–17], it is still a challenge to clarify their actual control mechanisms, especially with respect to biological interplay.

Coastal wetlands, located in the transition zone between the land and the sea, include multiple types such as estuary, delta, saltmarsh, mangrove, and seagrass wetland [18]. They provide many valuable ecosystem services (e.g., flood protection, erosion control, and wildlife habitat) and are thus very important for human well-being [19]. Planktonic bacteria and archaea play an important role in driving nutrient biogeochemical cycles and maintaining ecological functions in aquatic ecosystems [20–22]. However, to the best of our knowledge, their biogeographic patterns are largely unknown in coastal wetlands including multiple types and at a large space scale. The existing studies are confined to a certain estuary [23], saltmarsh [24], or mangrove, and the subjects are mainly focused on soil microorganisms. Therefore, it is necessary to understand biogeographic patterns of planktonic bacteria and archaea and the processes shaping their community assembly in the coastal wetlands.

Twenty-one China’s coastal wetlands were investigated to understand the biogeographic patterns and control mechanisms of their planktonic bacteria and archaea. The wetlands stretch across nearly 20° of latitude from the northernmost of Liaoning Province to the southernmost of Hainan Province, including reservoir, river, estuary, delta, lagoon, bay, marsh, and mangrove wetland. The inlet and outlet, open water area, and vegetation area of wetland are considered to understand microbial regional spatial difference. Our hypothesis is that planktonic bacteria and archaea co-occurrence to shape their biogeographic patterns in coastal wetlands. Using 16S rRNA amplicon sequencing and quantitative PCR (qPCR), the community composition and abundance of planktonic bacteria and archaea were investigated. In addition, the physical and chemical parameters of wetland waters were measured, and null model and correlation-based network are carried out to understand the underlying processes and the bacteria-archaea co-occurrence relationship. The main aims of this study are: (1) to understand planktonic bacteria and archaea community composition and controlling factors; (2) to discern the relative importance of deterministic and stochastic processes on the biogeographic patterns, and (3) to discover planktonic bacteria-archaea co-occurrence patterns in various coastal wetlands. Therefore, the study helps to improve our understanding of aquatic microbial ecology in coastal wetlands.

## Methods

### Study area and sampling

**Fig. 1:**
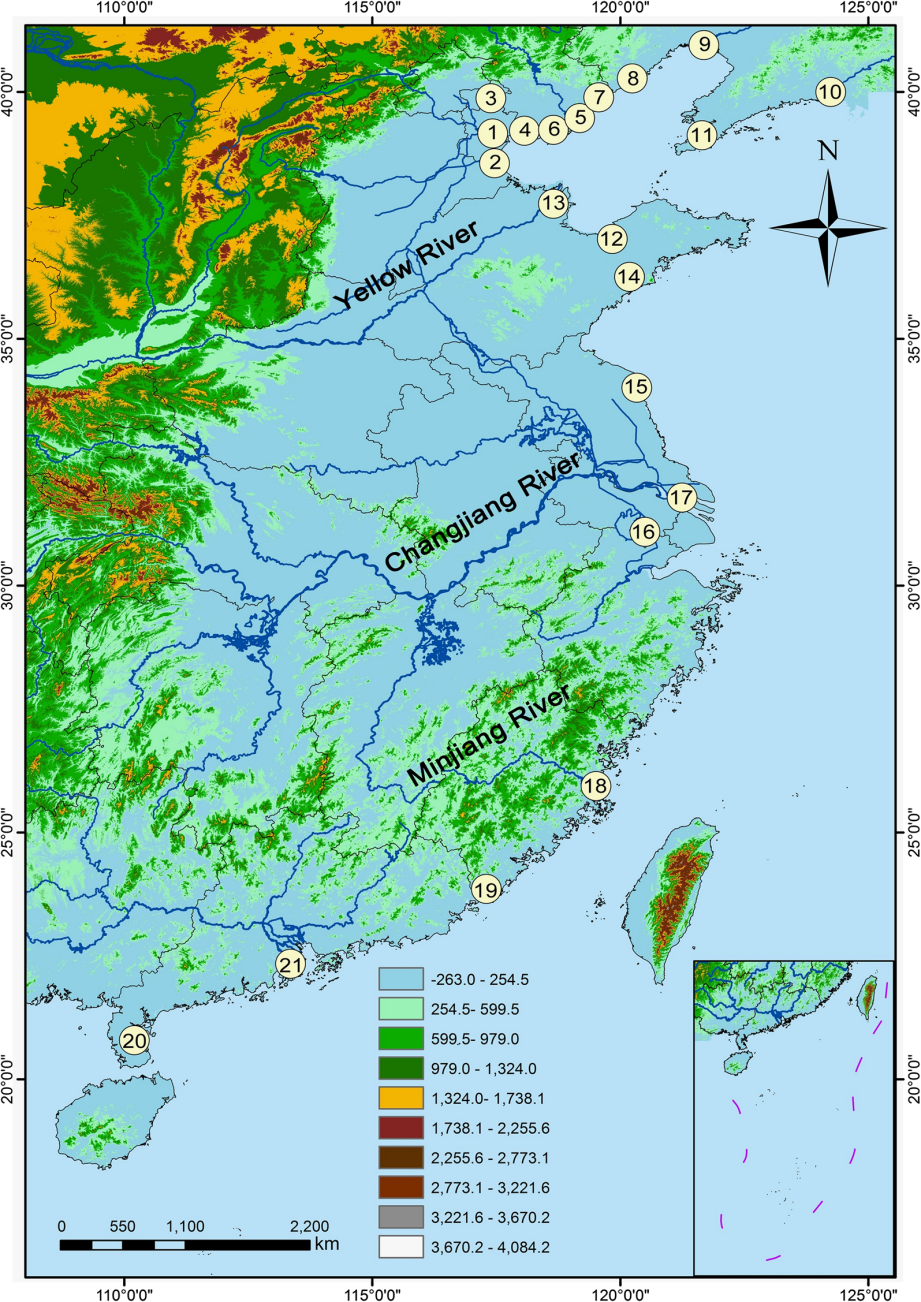
A map showing sampling areas in China’s coastal wetlands

China’s coastal wetlands have subtropical and temperate monsoon climates. The average annual temperature increases gradually from north to south, but in summer the temperature difference is small. Twenty-one coastal wetlands were selected for this study (Fig. 1; Additional file 1: Table S1). Their types are diverse and include four reservoir, one lagoon, four estuary, five river, two marsh, one bay, one delta, and three mangrove wetlands. Except the mangrove wetlands, the main vegetation of wetlands are *Phragmites communis* and *Acorus calamus*. Samples were collected from the inlet, outlet, and wetland waters. For the wetlands, surface and bottom waters were collected in open water area, while only surface water was collected in vegetated area. As such, a total of 101 water samples were collected during June and August 2019. Water temperature (WT), dissolved oxygen (DO), chlorophyll *a* (Chl *a*), salinity, and pH were measured directly using professional plus multi-parameter probe (YSI, EXO1, America) with precorrection in situ. For analyses of nutrients, the water samples were filtered through 0.45 μm filters (GF/F-Whatman, UK) within 12 h after sampling, and the filtered waters were stored in 100 ml polyethylene bottles at 4 °C. Particulate organic carbon (POC) and nitrogen (PON) were collected with pre-combusted (450 °C, 8 h) 0.70 μm filters (GF/F-Whatman, UK). Bacterial and archaeal assemblages were collected by 0.22 μm sterilized filter membrane (MF-Millipore, USA) with diaphragm vacuum pump (GM-1.0A, China), and then stored at − 20 °C until DNA extraction.

### Physicochemical analysis

Total nitrogen (TN), ammonium nitrogen (NH_4_-N), nitrate nitrogen (NO_3_-N), nitrite nitrogen (NO_2_-N), total phosphorus (TP), phosphate phosphorus (PO_4_-P), and dissolved silicon (DSi) were quantified by Skalar (San++, Netherlands) with detection limit of 0.01 mg L^−1^ for TN and NO_3_-N, 0.005 mg L^−1^ for NO_2_-N, 0.02 mg L^−1^ for NH_4_-N, TP, PO_4_-P, and DSi, respectively. POC and PON were measured by Elemental analyzer (Vario EL III, Germany). Dissolved organic carbon (DOC) was determined by a TOC analyzer (Aurora 1030, USA).

### DNA extraction and quantitative PCR

Water microbial DNA extraction was carried out using the E.Z.N.ATM Water DNA Kit (OMEGA, USA) according to manufacturer’s protocols. The primer sets 906F/1062R [25] and 1106F/1378R [26] were respectively used for bacterial and archaeal 16S rRNA gene amplification. 16S rRNA gene abundance of planktonic bacteria and archaea in each sample was determined by qPCR using Bio-rad Fax (CFX ConnectTM, USA). Triplicate amplifications were conducted in a 20 μl reaction system containing 12.5 μl of Ultra SYBR Mixture (2 ×), 1.0 μl of each primer (10 nM), 10 ng of DNA template, and 4.5 μl ddH_2_O. The thermal cycling was performed in two steps: initial denaturation was at 95 °C for 10 min, 40 cycles of 95 °C for 15 s, 60 °C for 1 min, and then melting curve program was 95 °C for 15 s, 60 °C for 1 min, 95 °C for 15 s, and 60 °C for 15 s. The standard curve was generated by tenfold dilution of plasmids (containing single-copy bacteria or archaea 16S rRNA gene, respectively) from 10^10^ to 10^4^ copies μl^−1^. The quantitative standard curve was built as bacteria 16S rRNA gene copies = − 2 × 10^10^ Ct + 5 × 10^11^ (R^2^ = 0.99) and archaea 16S rRNA gene copies = − 9 × 10^8^ Ct + 3 × 10^10^ (R^2^ = 0.82), respectively, where Ct was the cycle threshold. 16S rRNA gene abundance was calculated based on the Ct value together with plasmid standard curve.

### 16S rRNA sequencing denoising, community composition and α-diversity analysis

Sequencing was performed on Illumina NovaSeq 6000 platform at Meige Technology Co., Ltd., Guangzhou, China. Raw fastq data were quality-filtered using Trimmomatic [27] to remove contaminating adaptors, low-quality ends of reads, and short length reads (< 200 bp). The overlapping paired-end reads with sequence mismatching < 5 bp and alignment similarity > 90% were merged using FLASH [28]. Operational taxonomic units (OTUs) were clustered in UPARSE software at 97% consistency level [29]. Singleton and doubleton OTUs representing sequencing errors were removed, and the representative OTUs with the highest occurrence frequency were assigned using Silva, RDP and Greengene [30]. To equalize sequencing depth, each sample was rarefied to the minimum sequencing depth, and sequence normalization was performed using MOTHUR v.1.33.3 [31]. Bacterial and archaeal raw data obtained have been deposited in NCBI SRA database with the accession numbers of PRJNA681135 and PRJNA674461. Phylogenetic analysis of community composition was performed in the R package, α-diversity including shannon_e was conducted with the “vegan” package v 2.0-4 in R [32].

### Quantification of ecological processes using null model

Null model can integrate phylogenetic and taxonomic β-diversity metrics to quantify the relative importance of stochastic and deterministic processes on microbial community assembly [33, 34]. Beta mean nearest taxon metric (βMNTD) was calculated using the picante R package iCAMP [35], and then null model was implemented to calculate the beta nearest taxon index (βNTI) [33, 36]. |βNTI| > 2 is interpreted as variable selection of deterministic processes, and |βNTI| < 2 indicates that community composition difference is the result of stochastic processes [37]. Further, the Bray–Curtis dissimilarities based Raup-Cricck metric (RC_bary_) was calculated. When |βNTI| < 2, RC_bary_ < − 0.95 and RC_bary_ > 0.95 indicate that community assembly is dominated by homogenizing dispersal and dispersal limitation respectively, and |RC_bray_| < 0.95 indicate the influence of the “non-dominant” fraction [14]. Niche breadth of bacteria and archaea calculated using the “spaa” libraries in R.

### Network analysis

Correlation-based network analysis has been extensively used for microbial interactions [38, 39], and the co-occurrence patterns illustrated by a network can capture important information in microbial ecology [40]. Co-occurrence networks were constructed using the “WGCNA” libraries in R 4.0.0, and were visualized with “Gephi 0.9.2” software. To reduce complexity, only OTUs with the total number above 62 across all samples and occurring in more than 30% of all samples were retained, meanwhile, removing the relationship by themselves. The pairwise Spearman’s correlations between OTUs were calculated, with a correlation coefficient > 0.7 and a *P *value < 0.05 (Benjamini and Hochberg adjusted) being considered as a valid relationship. The network-level topological features of a network was calculated.

### Data analysis

Spearman’s correlation analyses between physicochemical factors were performed using the core function in the ‘corrplot’ package. Mantel test was used for correlational analysis between environmental factors and microbial community composition according to the *r* and significance level *P*-value of the two matrices. All statistical analyses were performed using R software (version 3.6.3). Variation partitioning analysis (VPA) was determined the proportion of specific environmental factors to explain the changes of community structure by using “vegan” libraries in R.

## Results

### Physicochemical parameters

**Fig. 2:**
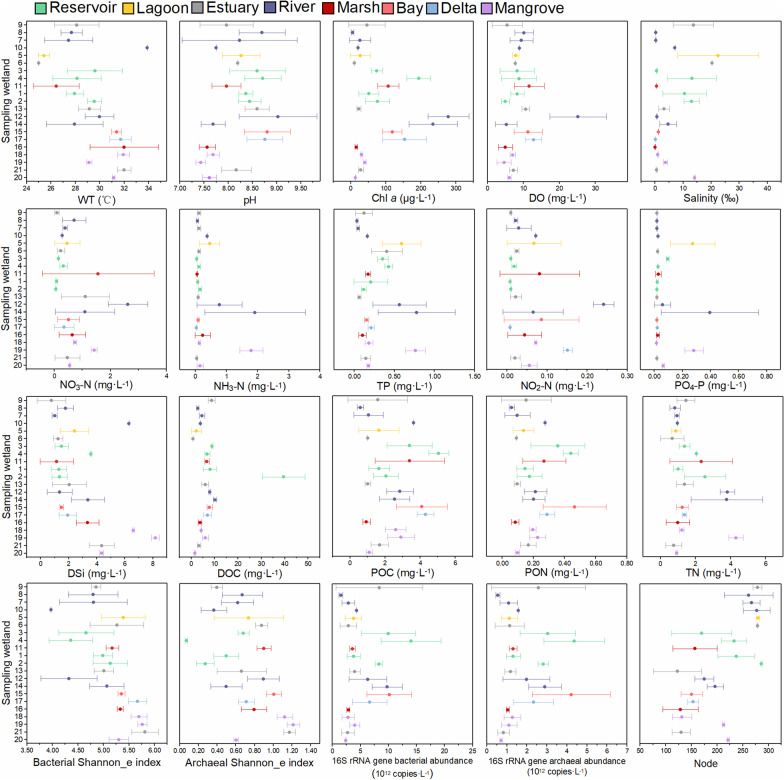
Spatial variations in physicochemical parameters, α-diversity index and abundance of planktonic bacteria and archaea, and node of sub-network topology. The different colors represent different wetland types. The relevant abbreviations are referred to the text

The WT ranged from 21.0 to 36.0 °C with an average of 29.0 °C and showed a weak increasing trend from the north to the south (Fig. 2). Salinity ranged from 0.00 to 36.00‰ with an average of 5.81‰ and was usually high in the north wetlands. Trophic level-related parameters ( including TN, TP, Chl *a*, POC, and PON) usually showed high values in the middle wetlands, whereas DSi seemed decreasing from the north to the south. Other physicochemical parameters had disorderly spatial variation. All these parameters did not show regular patterns based on the wetland types (Fig. 2).

### Planktonic bacteria and archaea 16S rRNA gene abundance, α-diversity and community composition

Bacteria 16S rRNA gene abundance was from 0.67 to 31.95 × 10^12^ copies L^−1^ with an average of 5.4 × 10^12^ copies L^−1^, and that of archaea was from 0.71 to 12.92 × 10^12^ copies L^−1^ with an average of 1.8 × 10^12^ copies L^−1^. They were usually high in the middle wetlands (Fig. 2). Shannon_e index of bacteria was higher than that of archaea, and both of them showed high values in the south wetlands.

**Fig. 3:**
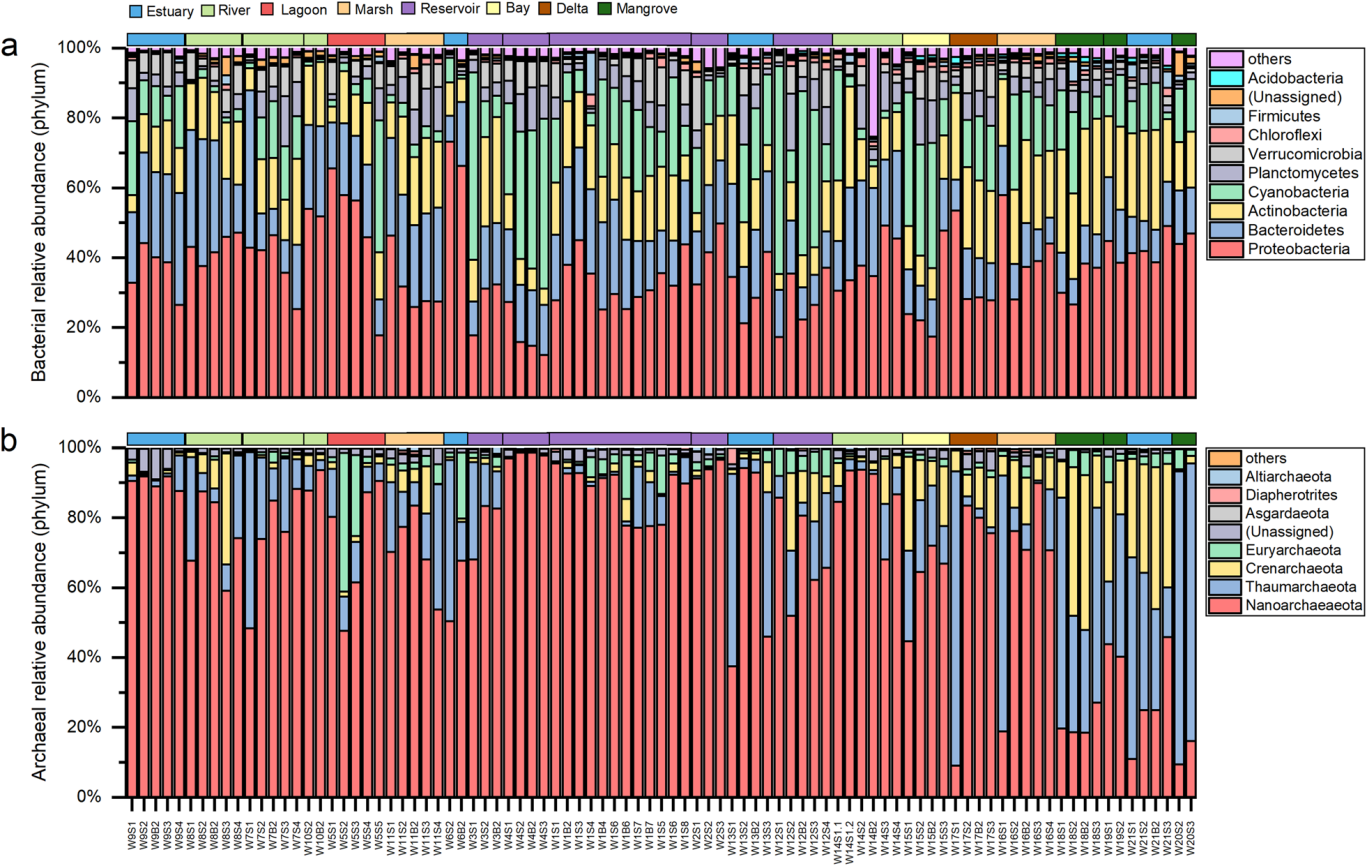
The composition of bacterial and archaeal community. **a** Planktonic bacteria; **b** Planktonic archaea. Different colors in the rectangle above each figure represent different wetland types. The x-axis labels mean sampling sites from north to south, and the details are referred to Additional file 1: Table S1

A total of 18,819,251 and 6,736,335 reads were obtained after quality control and then clustered into 53,503 and 21,350 OTUs in bacteria and archaea, respectively. All bacteria OTUs yielded by high-throughput sequencing were clustered into 63 phyla, whereas archaea OTUs only assembled 9 phyla. For bacteria, Proteobacteria and Bacteroidetes were top 2 phyla, and the next phyla were followed by Actinobacteria and Cyanobacteria with relative abundance more than 10% (Fig. 3a). For archaea, Nanoarchaeaota, Thaumarchaeaota, Crenarchaeota and Euryarchaeota were dominant phyla (Fig. 3b). From the north to the south, archaea community structure showed larger variation than that of bacteria (Fig. 3, Additional file 1: Table S1).

### Planktonic bacteria and archaea co-occurrence networks

**Fig. 4:**
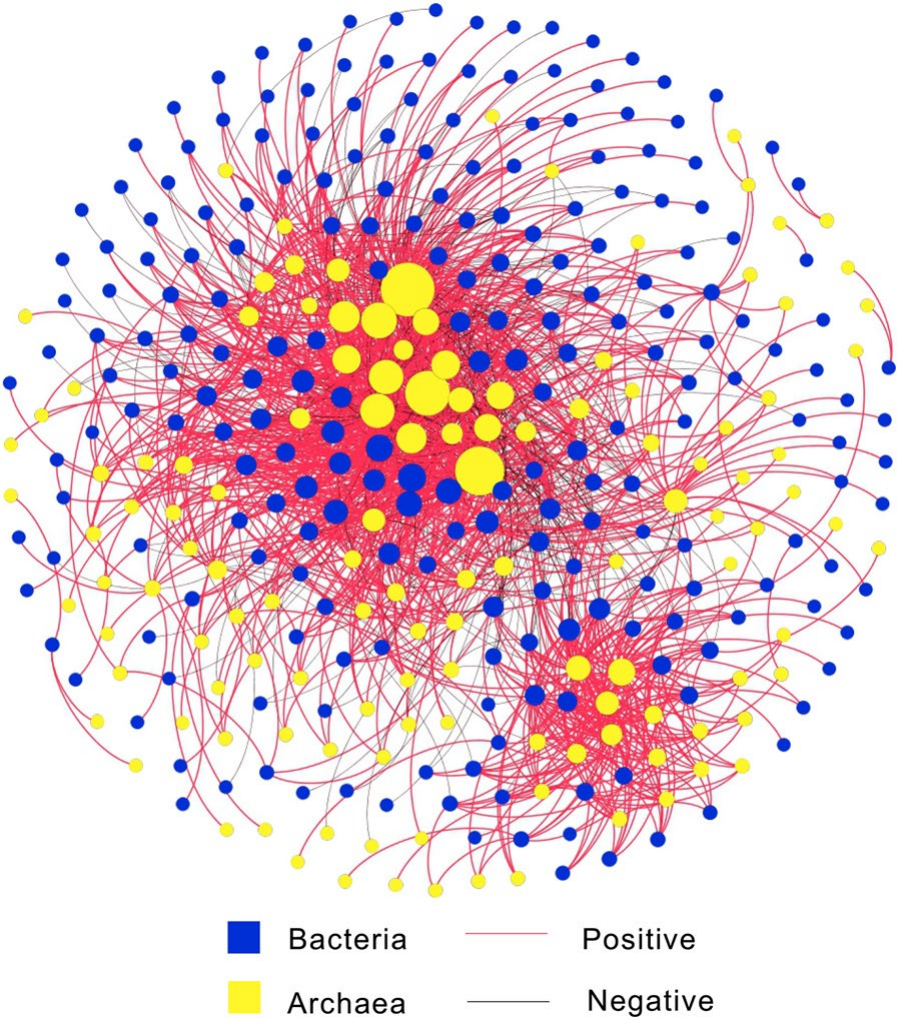
Co-occurrence networks of the planktonic bacteria and archaea. The size of each node is proportional to the number of connections

The co-occurrence networks consisted of 357 nodes and 1687 edges (Fig. 4). Most of them were positive correlation. Planktonic archaea were in a core position and had more contact with planktonic bacteria. Sub-networks of each water sample were generated, and the nodes of sub-networks of each wetland decreased with the increasing latitude (Fig. 2).

### Bate mean nearest taxon metric

Only 19.4% of the βNTI scores for 16S rRNA OTUs derived from bacteria community were in the range of − 2 to + 2, indicating that deterministic processes dominated planktonic bacteria community assembly in the wetlands (Fig. 6a). However, planktonic archaea βNTI scores in the range of − 2 to + 2 accounted for 59.3% of the total, which was indicative of stochastic processes to be greater than deterministic processes in their community assembly in the wetlands (Fig. 6b). For stochastic processes, dispersal (homogenizing), drift (acting alone), and dispersal (limitation combined with drift) was divided based on the RC_bary_, and the dispersal (limitation combined with drift) was prominent in planktonic bacteria (19.0%) and archaea (53.4%) community assembly.

**Fig. 5:**
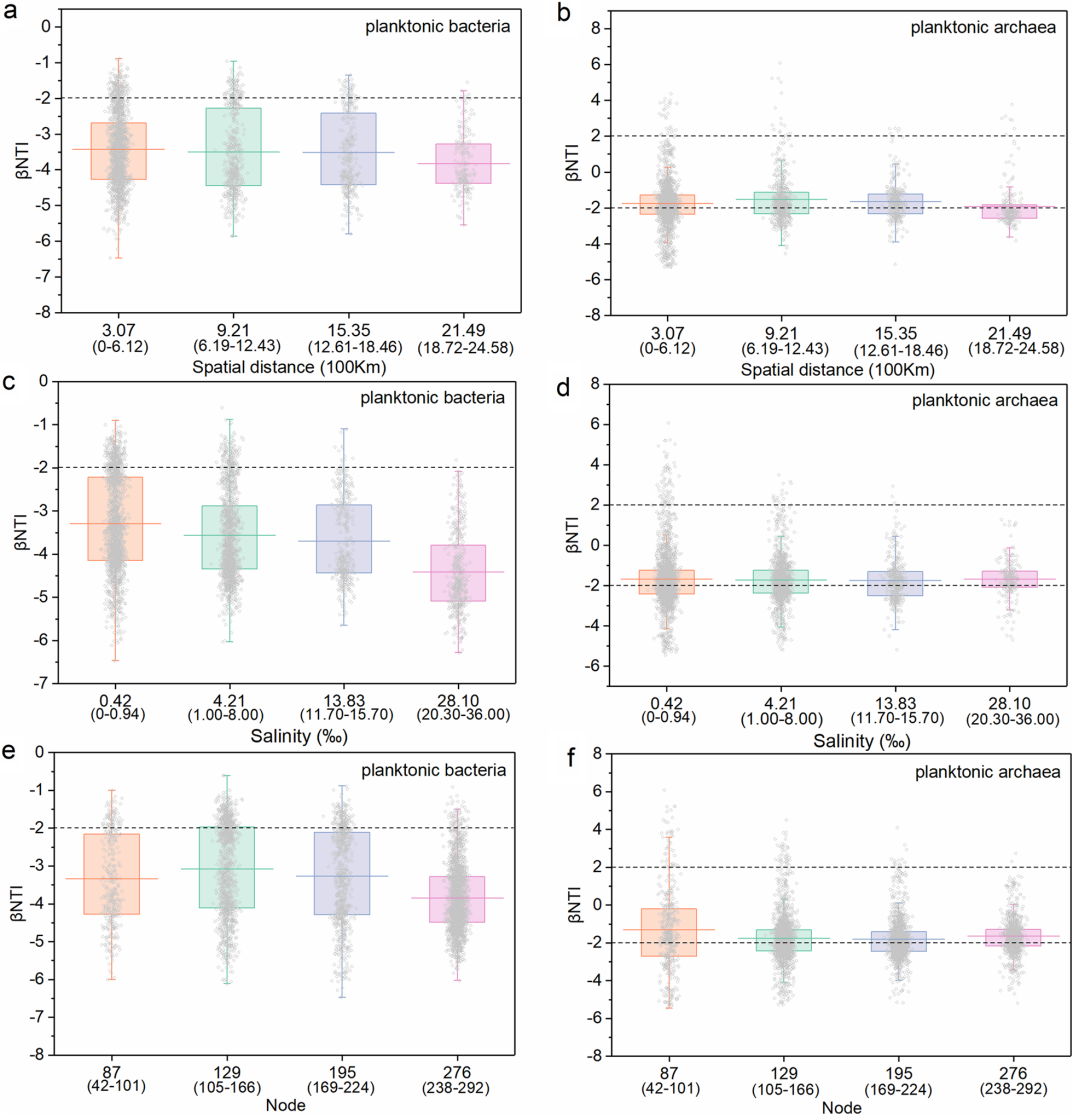
Beta Nearest Taxon Index (βNTI) in China’s coastal wetlands along geographical distance (**a**, **b**), salinity (**c**, **d**), and node (**e**, **f**), respectively. Horizontal dashed lines indicate βNTI values of − 2 and 2

**Fig. 6:**
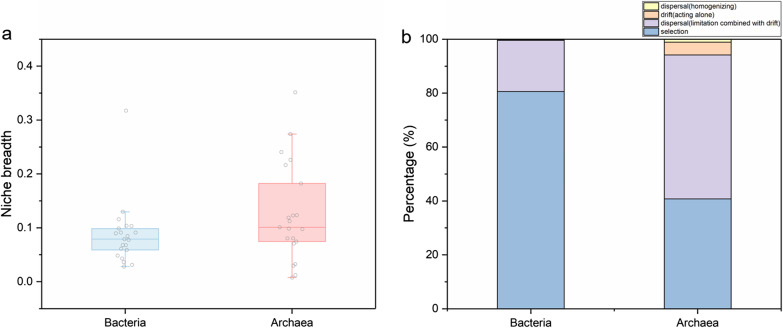
**a** Box plots showing niche breadths of planktonic bacteria and archaea; the boxes indicate the 25th to 75th percentiles and the whiskers indicate the 10th and 90th percentiles. The central lines indicate the median. **b** Relative importance of assembly processes shaping the planktonic bacterial and archaeal communities

The βNTI were grouped according to the spatial distance, salinity, and nodes, respectively (Fig. 5). The planktonic bacteria βNTI showed a decrease with an increase in those parameters, indicating an enhancement of deterministic processes on their community assembly. However, this phenomenon was not found for planktonic archaea in the wetlands. For the niche breadth, planktonic archaea showed greater value than planktonic bacteria (Fig. 6a).

### Relationship between community composition and environmental factors

**Fig. 7:**
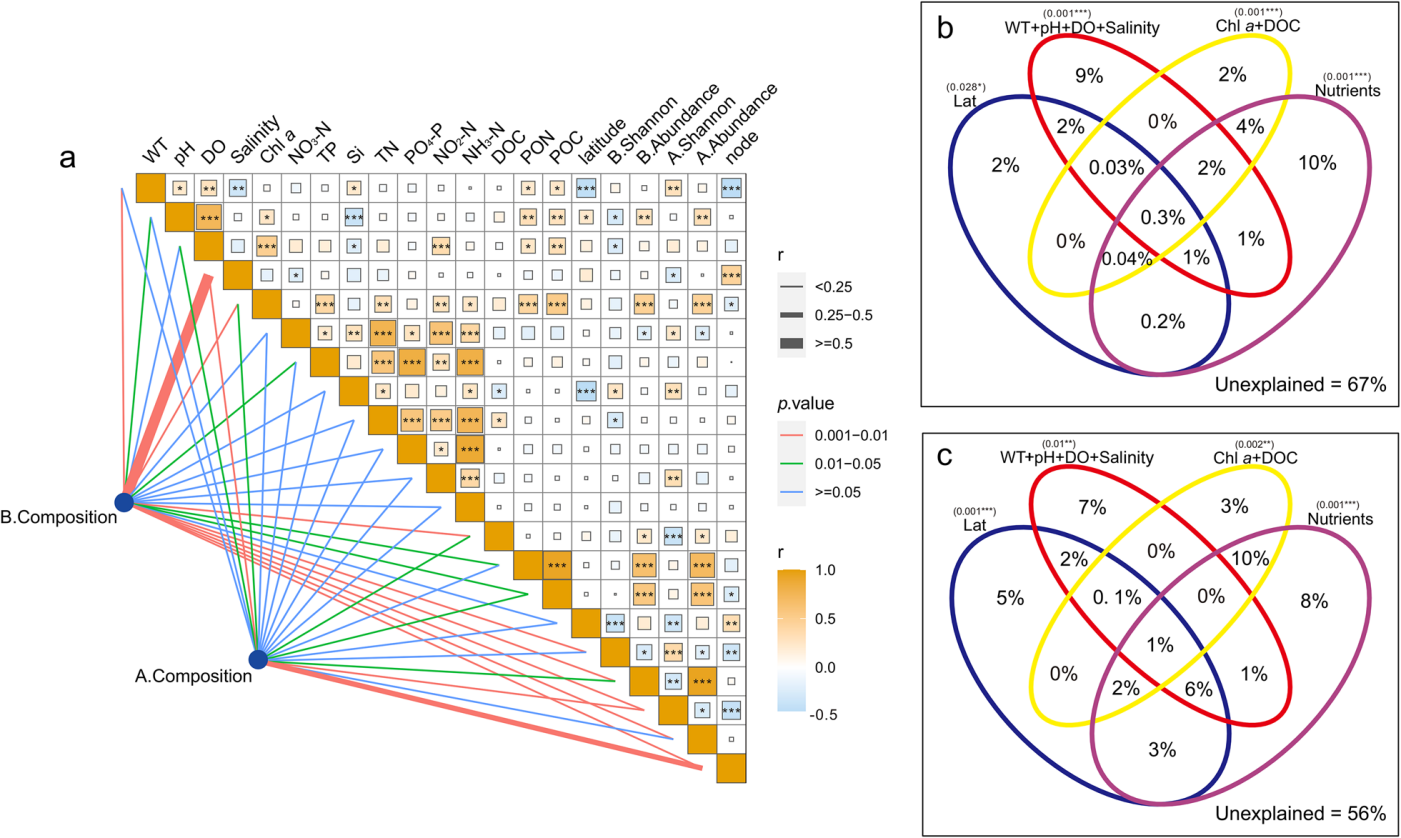
**a** Correlations between physicochemical factors and community characteristic parameters of planktonic bacteria and archaea. Pairwise comparisons of physicochemical factors are displayed with a color gradient to denote Spearman’s correlation coefficients. Community composition is related to each environmental factor by performing a Mantel test. Significance level: *p* < 0.001***; *p* < 0.01**; *p* < 0.05*. **b** Variation partitioning analyses (VPA) of planktonic bacteria community. **c** VPA of planktonic archaea community. The full name of each parameter is referred to the text

**Fig. 8:**
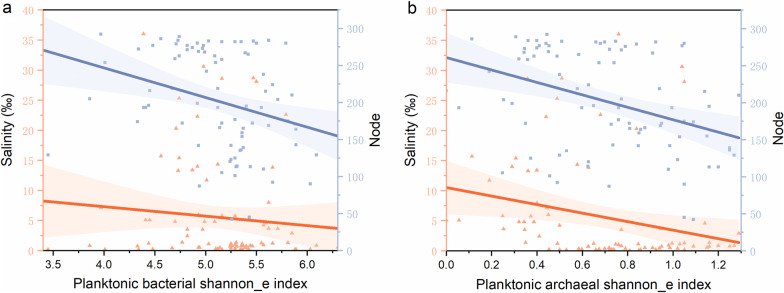
**a** Bacterial shannon_e index vs salinity and bacterial shannon_e index vs node of sub-network topology; the regression (blue): y = − 40.02x + 406.80, *r*^2^ = 0.11; the regression (orange): y = − 1.57x + 13.56, *r*^2^ = 0.01. **b** Archaeal shannon_e index vs salinity and archaeal shannon_e index vs node of sub-network topology; the regression (blue): y = − 83.95x + 260.98, *r*^2^ = 0.15; the regression (orange): y = − 7.08x + 10.50, *r*^2^ = 0.07. The blue-shaded and orange-shaded sections show 95% confidence intervals

Variation partitioning analysis indicated the unexplained of bacterial community composition and archaeal community composition was accounted for 67% and 56%, respectively (Fig. 7b, c). Salinity and node were found to be key influencing factors based on mantel test (Fig. 7a) and showed significant negative correlation with shannon_e index of bacteria and archaea, respectively (Fig. 8).

## Discussion

### Mechanisms underlying geographical patterns of planktonic bacteria and archaea in the coastal wetlands

There are different contributions of stochastic and deterministic processes to planktonic bacteria and archaea community assembly in the coastal wetlands along geographical distance. The deterministic selection shapes planktonic bacteria community, while stochastic processes regulate planktonic archaea community assembly (Fig. 5a, b). For the latter, the dispersal combined with drift was suggested to be a dominance in the stochastic processes, and the relative importance of dispersal and ecological drift is stronger than deterministic selection (Fig. 6b). Few studies are about planktonic archaea community assembly across complex coastal wetlands, and a stochastic-dominated archaea assembly in coastal waters of Northern Zhejiang has been reported recently [41]. The contrasting mechanisms for planktonic bacteria and archaea assembly may be attributed to their different niche breadth. The wider niche breadth is less affected by environmental selection [42], and planktonic archaea are found to be wider niche breadth than planktonic bacteria (Fig. 6a) and thus tend to be stochastic regulation. With an increase in geographical distance, deterministic-dominated processes underlying the planktonic bacteria and archaea assembly tend to strengthen (Fig. 5a, b), probably because an excessive increase in geographical distance would weaken the ability of microbial dispersal and drift [43], and local deterministic selection gradually dominates community assembly.

In addition, there were two clusters with significant habitat differences: the inlet and outlet waters and the wetland waters (Additional file 1: Fig. S1), suggesting that local ecological niche plays a crucial role in shaping planktonic microbial community composition over a short distance. The inlet and outlet waters are lotic, while the wetland waters have a higher hydraulic retention time. This can result in their different microbial communities because hydrologic conditions have been reported to control nutrient biogeochemical cycling [44] and shape microbial composition [45]. In addition, wetland vegetation maintain ecosystem structure and function by recycling nutrients, attenuating flow velocities, releasing oxygen and organic carbon, and stabilizing the sediment [46, 47]. This helps to shape the unique environmental conditions of wetlands, where bacteria community structure in the surface waters is similar to that in the bottom waters due to the shallow vertical depth (< 2 m). Different from planktonic bacteria, the similarity of planktonic archaea community structure is generally low, but cluster analysis still show dynamic difference (Additional file 1: Fig. S1). All these differences are attributed to their different environmental factors (Additional file 1: Fig. S2).

### Deterministic environmental factors for planktonic bacteria and archaea community in the coastal wetlands

In China’s coastal wetlands, salinity is a key factor in shaping planktonic bacteria community composition; whereas a key determinant for planktonic archaea community assembly is not found (Fig. 7a), which is consistent with its stochastic-dominanted regulation. Salinity has been considered as a stress factor of bacteria community and can affect bacteria respiratory activity, membrance polarization and integrity, and DNA and RNA contents [48, 49]; therefore, salinity has been reported to be a major determinant affecting the biogeographic pattern of bacterial communities across lake and marine ecosystems [50]. For planktonic archaea, although Crenarchaeota, Euryarchaeota and Thaumarchaeota are well known as the dominant archaea in brackish and pond sediment [51, 52], Nanoarchaeaeota are the top one in this study (Fig. 3), indicating their pervasiveness in the coastal wetland waters. In general, deterministic environmental factors only explain less than 44% of planktonic bacteria and archaea community composition in the coastal wetlands (Fig. 7b, c), and other factors such as biological interaction might play an important role in their community assembly.

The abundance of planktonic bacteria and archaea is mainly determined by TP, Chl *a*, POC and PON in the coastal wetlands (Fig. 7a). It is reasonable because they are nutrient-related environmental factors, and planktonic bacteria and archaea need them to support their growths. These factors are usually positively correlated from each other (Fig. 7a), indicating the importance of phytoplankton as a primary producer to some degree.

### Effect of planktonic bacteria-archaea co-occurrence on their biogeographic patterns in the coastal wetlands

To the best of our knowledge, this is the first study that used network analysis to explore planktonic bacteria-archaea co-occurrence in the coastal wetlands. The large number of correlations imply that bacteria and archaea coexist in the same habitat and are functional interdependencies (Fig. 4). Nanoarchaeaeota are obligate symbiont with chemolithotrophic Crenarchaeon *Ignicoccus hospitalis* that is a sulfur reducer [53]. As such, Nanoarchaeaeota should be involved in sulfur cycling along with Proteobacteria. In addition, Thaumarchaeota and Crenarchaeota are ammonia-oxidizing archaea containing amoA enzyme and can oxidize ammonia to nitrite during nitrification [54, 55]; as such, they will play an important role in nitrogen cycling together with Proteobacteria and Bacteroidetes. These interactions among the specific species finnally result in the tight coorelations in the network analysis although variations in co-occurrence patterns that are derived from a topology-based system approach have been reported not to reflect true inter taxa correlations and only to reveal partial complex interaction within a microbial community [56–58]. In addition, the nodes of sub-networks rather than salinity showed greater significant relationship with the shannon_e index of planktonic bacteria and archaea (Fig. 8), and their community composition also exhibited significant correlation with their biological parameters (i.e., shannon_e index, nodes of sub-networks, and 16S rRNA gene abundance) (Fig. 7a). All these demonstrate that planktonic bacteria-archaea co-occurrence play an important role in shaping their biogeographic patterns in the coastal wetlands.

## Conclusion

Coastal wetlands are special and complex ecosystems. Planktonic bacteria and archaea in China’s coastal wetlands have different biogeographic patterns and control mechanisms. Deterministic selection shapes the planktonic bacteria community structure, and salinity is a main controlling factor for their community assembly. Stochastic processes regulate the planktonic archaea community structure, being consistent with the fact that planktonic archaea have a larger niche breadth than planktonic bacteria. Planktonic bacteria and archaea co-occur, and their co-occurrence play an important role in their community assembly. This study helps to improve an understanding about biogeographic patterns of planktonic microbes, and thus provides new insight into studying underlying mechanisms of microbial biogeography in coastal wetlands.

## Supplementary Information

**Additional file 1**. **Fig. S1.** Cluster analysis of planktonic bacteria and archaea based on community composition similarity. **Fig. S2.** Correlations between physicochemical factors and community characteristic parameter of planktonic bacteria and archaea. **Table S1.** Sampling information of twenty-one China's coastal wetlands.

## Data Availability

The datasets analysed during the current study are available in NCBI SRA database with the accession numbers of PRJNA681135 and PRJNA674461.
