# Supplementary material for: Co-occurrence of planktonic bacteria and archaea affects their biogeographic patterns in China’s coastal wetlands
Source: Environ Microbiome. 2021 Oct 19;16:19. doi: 10.1186/s40793-021-00388-9 (PMC8527667; doi:10.1186/s40793-021-00388-9)
Supplement: Supplementary file 1 — Additional file 1. Fig. S1. Cluster analysis of planktonic bacteria and archaea based on community composition similarity. Fig. S2. Correlations between physicochemical factors and community characteristic parameter of planktonic bacteria and archaea. Table S1. Sampling information of twenty-one China's coastal wetlands. [file 40793_2021_388_MOESM1_ESM.docx]

**Supplementary information**

Co-occurrence of planktonic bacteria and archaea affects their biogeographic patterns in China’s coastal wetlands

Baoli Wang^1, 2 *^, Na Liu^1^, Meiling Yang^1*^, Lijia Wang^1^, Xia Liang^3^, Cong-Qiang Liu^1, 2^

^1^Institute of Surface-Earth System Science, School of Earth System Science, Tianjin University, Tianjin 300072, China

^2^Critical Zone Observatory of Bohai Coastal Region, Tianjin University, Tianjin 300072, China

^3^State Key Laboratory of Estuarine and Coastal Research, East China Normal University, Shanghai 200244, China

* Corresponding author: Baoli Wang (Email: [baoli.wang@tju.edu.cn](mailto:baoli.wang@tju.edu.cn)) and Meiling Yang (meilingyang@tju.edu.cn), Institute of Surface-Earth System Science, School of Earth System Science, Tianjin University, Tianjin 300072, China. Tel: +86 (0)22 27405053; Fax: +86 (0)22 27405051.

**This file includes** **Figure S1 and S2 and Table S1.**


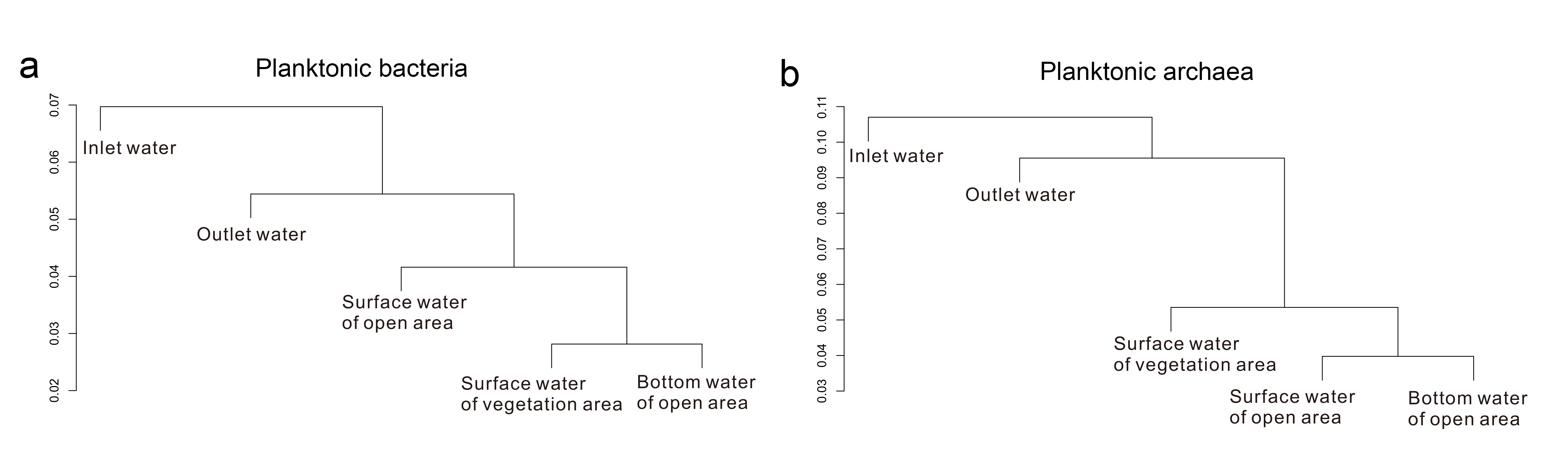


**Fig. S1** Cluster analysis of planktonic bacteria (a) and archaea (b) based on community composition similarity.


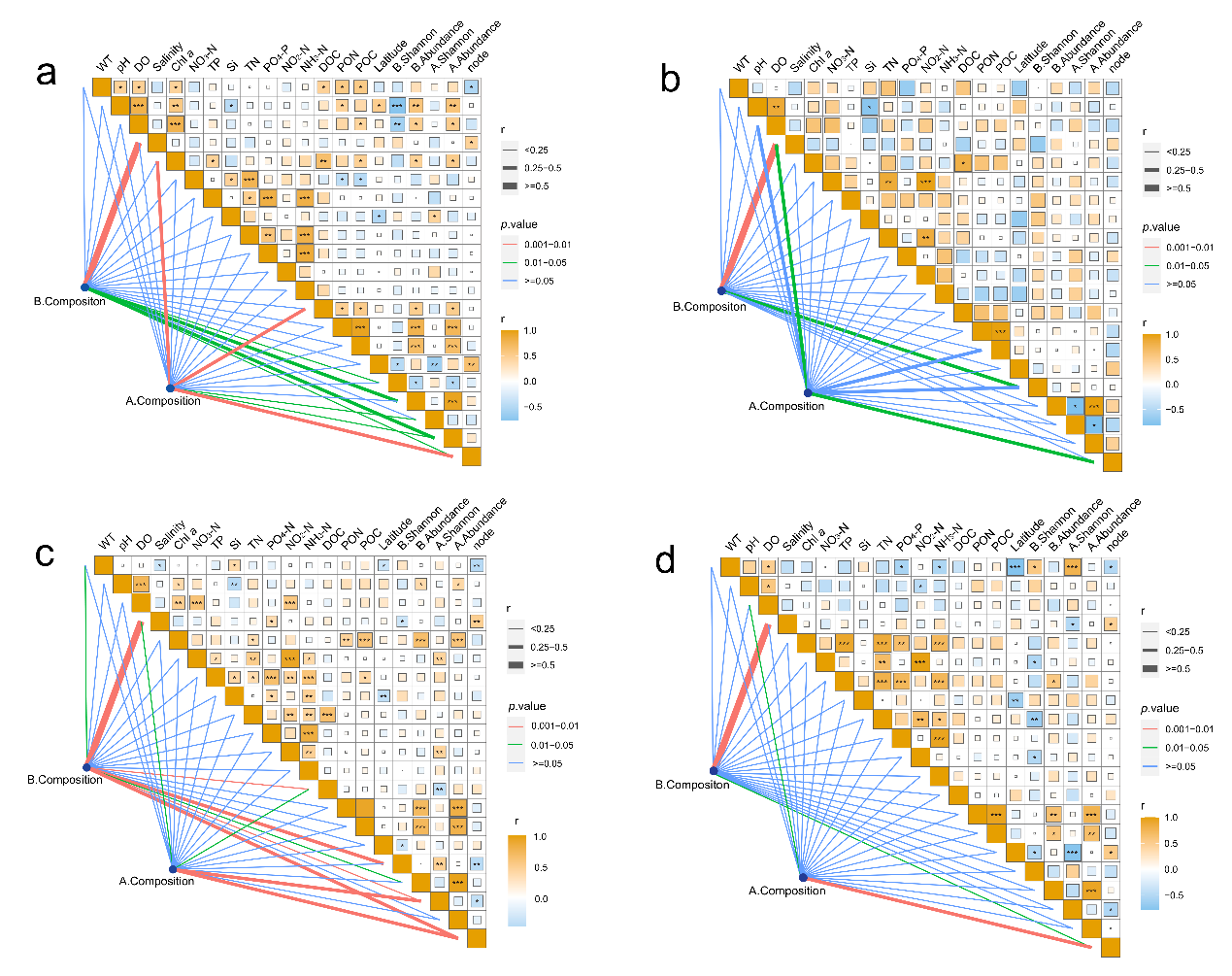


**Fig. S2** Correlations between physicochemical factors and community characteristic parameters of planktonic bacteria and archaea. Inlet water (a); Outlet water (b); Surface and bottom water of open area in wetland (c); Surface water of vegetation area in wetland (d).

**Supplementary Table S1** Sampling information of twenty-one China’s coastal wetlands

| **Coastal**  **wetland** | **Sampling**  **areas** | **Sampling**  **sites** | **Sampling**  **time** | **Longitude** | | **Latitude** | | **Location** | **Type** | | **Vegetation** | |
| --- | --- | --- | --- | --- | --- | --- | --- | --- | --- | --- | --- | --- |
|  | Inlet | W1S1; W1S8 |  |  |  | |  | |  |  | |  |
| Beidagang | Wetland | W1S2; W1B2; W1S3; W1S4, W1B4;  W1S6, W1B6; W1S7, W1B7  W1D2; W1D3 | 2019.06.29 | 117°23'55" | 38°46'2" | | Tianjin, China | | Reservoir | *Phragmites communis* | |  |
|  | Outlet | W1S5 |  |  |  | |  | |  |  | |  |
|  | Inlet | W2S1 |  |  |  | |  | |  |  | |  |
| Nandagang | Wetland | W2S2, W2B2; W2S3; W2B3  W2D2; W2D3 | 2019.07.02 | 117°29'28" | 38°32'33" | | Cangzhou, Hebei province, China | | Reservoir | *Phragmites communis* | |  |
|  | Inlet | W3S1 |  |  |  | |  | |  |  | |  |
| Qilihai | Wetland | W3S2; W3B2  W3D2 | 2019.07.03 | 117°28'58" | 39°17'2" | | Tianjin, China | | Reservoir | *Phragmites communis* | |  |
|  | Inlet | W4S1 |  |  |  | |  | |  |  | |  |
| Caofeidian | Wetland | W4S2; W4B2; W4B3  W4D2; W4D3 | 2019.07.04 | 118°22'19" | 39°11'9" | | Tangshan, Hebei province, China | | Reservoir | *Phragmites communis* | |  |
| Qilihai | Inlet | W5S1; W5S4; W5S5 |  |  |  | |  | |  |  | |  |
|  | Wetland | W5S2  W5D2 | 2019.07.07 | 119°16'5" | 39°34'46" | | Qinhuangdao, Hebei province, China | | Lagoon | *Phragmites communis* | |  |
|  | Outlet | W5S3 |  |  |  | |  | |  |  | |  |
| Luan River Estuary | Wetland | W6S2; W6B2  W6D2 | 2019.07.07 | 119°15'32" | 39°25'8" | | Qinhuangdao, Hebei province, China | | Estuary | *Phragmites communis* | |  |
|  | Inlet | W7S1 |  |  |  | |  | |  |  | |  |
| Dashi Riverside | Wetland | W7S2; W7B2; W7S3 | 2019.07.07 | 119°44'7" | 39°59'20" | | Qinhuangdao, Hebei province, China | | River | *Phragmites communis* | |  |
|  | Outlet | W7S4 |  |  |  | |  | |  |  | |  |
|  | Inlet | W8S1 |  |  |  | |  | |  |  | |  |
| Suizhong | Wetland | W8S2; W8B2; W8S3; W8B3  W8D2; W8D3 | 2019.07.09 | 120°21'14" | 40°20'46" | | Huludao, Liaoning province, China | | River | *Phragmites communis*  *Nymphaea tetragona* | |  |
|  | Outlet | W8S4 |  |  |  | |  | |  |  | |  |
|  | Inlet | W9S1 |  |  |  | |  | |  |  | |  |
| Liao River Estuary | Wetland | W9S2; W9B2; W9S3; W9B3  W9D2; W9D3 | 2019.07.10 | 121°46'38" | 40°56'46" | | Panjin, Liaoning province, China | | Estuary | *Phragmites communis* | |  |
|  | Outlet | W9S4 |  |  |  | |  | |  |  | |  |
| Yalvjiang | Wetland | W10S2  W10D2 | 2019.07.12 | 123°38'5" | 39°55'26" | | Dandong, Liaoning province, China | | River | *Phragmites communis* | |  |
|  | Inlet | W11S1 |  |  |  | |  | |  |  | |  |
| Qianguan | Wetland | W11S2; W11B2; W11S3; W11B3  W11D2; W11D3 | 2019.07.13 | 121°39'45" | 39°2'7" | | Dalian, Liaoning province, China | | Marsh | *Phragmites communis* | |  |
|  | Outlet | W11S4 |  |  |  | |  | |  |  | |  |
|  | Inlet | W12S1 |  |  |  | |  | |  |  | |  |
| Hetao | Wetland | W12S2; W12B2; W12S3; W12B3  W12D2; W12D3 | 2019.07.18 | 119°54'56" | 37°12'10" | | Laizhou, Shandong province, China | | River | *Acorus calamus* | |  |
|  | Outlet | W12S4 |  |  |  | |  | |  |  | |  |
|  | Inlet | W13S1 |  |  |  | |  | |  |  | |  |
| Yellow River Estuary | Wetland | W13S2; W13B2; W13S3; W13B3  W13D2; W13D3 | 2019.07.19 | 119°1'25" | 37°45'46" | | Dongying, Shandong province, China | | Estuary | *Phragmites communis* | |  |
|  | Inlet | W14S1-1; W14S1-2 |  |  |  | |  | |  |  | |  |
| Shaohai | Wetland | W14S2; W14B2; W14S3; W14B3  W14D2; W14D3 | 2019.07.21 | 120°6'4" | 36°15'17" | | Jiaozhou, Shandong province, China | | River | *Phragmites communis* | |  |
|  | Outlet | W14S4 |  |  |  | |  | |  |  | |  |
|  | Inlet | W15S1 |  |  |  | |  | |  |  | |  |
| Dandinghe | Wetland | W15S2; W15B2; W15S3; W15B3  W15D2; W15D3 | 2019.07.22 | 120°31'35" | 33°36'28" | | Yancheng, Jiangsu province, China | | Bay | *Acorus calamus* | |  |
|  | Inlet | W16S1 |  |  |  | |  | |  |  | |  |
| Xixi | Wetland | W16S2; W16B2; W16S3; W16B3  W16D2; W16D3 | 2019.07.24 | 120°4'25" | 30°16'15" | | Hangzhou, Zhejiang province, China | | Marsh | *Lemna minor*  *Eichhornia crassipes* | |  |
|  | Outlet | W16S4 |  |  |  | |  | |  |  | |  |
|  | Inlet | W17S1 |  |  |  | |  | |  |  | |  |
| Changjiang Delta | Wetland | W17S2; W17B2; W17S3; W17B3  W17D2; W17D3 | 2019.08.15 | 121°13'56" | 31°44'1" | | Shanghai, China | | Delta | *Phragmites communis*  *Acorus calamus* | |  |
|  | Inlet | W18S1 |  |  |  | |  | |  |  | |  |
| Minjiang Estuary Mangrove | Wetland | W18S2; W18B2; W18S3  W18D2; W18D3 | 2019.08.17 | 119°37'41" | 26°1'26" | | Fuding, Fujian, China | | Mangrove  Estuary | Mangrove | |  |
|  | Inlet | W19S1 |  |  |  | |  | |  |  | |  |
| Zhangjiang Estuary Mangrove | Wetland | W19S2  W19D2; W19D3 | 2019.08.19 | 117°25'21" | 23°55'8" | | Zhangzhou, Fujian, China | | Mangrove  Estuary | Mangrove | |  |
| Zhanjiang Mangrove | Wetland | W20S2; W20S3  W20D2; W20D3 | 2019.08.21 | 110°18'54" | 21°6'22" | | Zhanjiang, Guangdong, China | | Mangrove | Mangrove | |  |
|  | Inlet | W21S1 |  |  |  | |  | |  |  | |  |
| Nansha | Wetland | W21S2; W21B2  W21D2; W21D3 | 2019.08.23 | 113°38'18" | 22°36'21" | | Zhongshan, Guangdong, China | | Estuary | *Phragmites communis* | |  |
|  | Outlet | W21S3 |  |  |  | |  | |  |  | |  |
